# Knowledge and attitude of emergency physician about the emergency management of tooth avulsion

**DOI:** 10.1186/s12903-018-0515-5

**Published:** 2018-04-02

**Authors:** Laila A. Bahammam

**Affiliations:** Endodontics Department, Faculty of Dentistry, King Abdulaziz University, P. O. Box 80209, Jeddah, 21589 Kingdom of Saudi Arabia

**Keywords:** Avulsion, Dental trauma, Emergency management, Traumatic dental injury, Physician, Attitude, Knowledge

## Abstract

**Background:**

The aim of the study was to evaluate the knowledge of ER physicians with different specialties, experience and hospital sectors for the management of avulsed teeth in the emergency rooms of eight major hospitals in Jeddah, Saudi Arabia. It also covers their attitude towards receiving further education on tooth avulsion management.

**Methods:**

A cross-sectional survey was conducted at the emergency rooms of eight hospitals in Jeddah from August to December 2015. A self-administered questionnaire consisting of 31 multiple choice questions assessing both knowledge and attitude was distributed to 150 physicians who were working in the ER departments.

**Results:**

Response rate was 81.33%. Data revealed that 45.9% of the respondents did not have prior knowledge about avulsion. Physicians working in military hospitals had better knowledge about the ER management of avulsion cases than physicians of public hospitals. 80.3% of participants showed willingness to replant the tooth, however, 65.3% would not do it by themselves. 42.6% of the physicians did not know the importance of extra-oral time. Milk was selected as the best transport media for avulsed tooth by 31.1% of the participants. Regarding physicians’ attitude, 95.1% showed interest in receiving information about the subject.

**Conclusion:**

This study revealed that the majority of ER physicians lack the knowledge needed to manage avulsions cases. Hence, educational programs are necessary for ER physicians to provide proper management for those cases.

**Electronic supplementary material:**

The online version of this article (10.1186/s12903-018-0515-5) contains supplementary material, which is available to authorized users.

## Background

Traumatic injuries to dentoalveolar tissues are considered the most serious oral health problem amongst children and adolescents. One of the types of traumatic dentoalveolar injury is tooth avulsion that is defined as total dislodgment of the tooth from its socket. It represents about 16% of all traumatic dentoalveolar injuries [1].

Maxillary central incisor is the most affected tooth [2, 3] due to their labial projection which make them more susceptible to facial trauma [2]. Moreover, these teeth have minimal resistance to frontal impact among children aged 7–9 years because of the lack of the periodontal ligament’s resiliency and the low mineralization of the surrounding bone [2]. Permanent anterior teeth are not only essential for aesthetics but also for phonetics, mastication and psychological health of young patients. Hence, immediate and appropriate management of the avulsed tooth by replantation is critical for long term prognosis, especially in children [2]. Failure to replant the tooth will lead to expensive, time-consuming, and complex multidisciplinary approaches [4]. When replantation is not possible, appropriate handling of the avulsed tooth will help to ensure long-term successful outcome [2]. Similarly, incorrect handling and replantation of avulsed tooth will lead to tooth resorption or ankyloses and result in poor prognosis [2].

The prognosis of tooth replantation is dependent on multiple factors; such as time of endodontic intervention, storage media, extra-alveolar period, type of retention employed, and type of drug prescribed. However, the most critical factor for an excellent prognosis is the preservation of viable periodontal ligament cells (PDL) covering the root, this can be established by storing the tooth in a suitable media such as milk, saliva or saline [2].

Tooth avulsion usually occurs because of road traffic accidents, falls and other physical impacts. It could happen in schools during sports or because of physical violence. In these situations, parents usually take their children to visit an emergency (ER) physician at the hospital to seek treatment. In such facilities, there is a lack of availability of a full-time dentist [5]. Therefore, to insure proper management of avulsed tooth, it is important for ER physicians to have sufficient knowledge about the emergency management of such cases [6].

There are many studies conducted in several countries that showed medical physicians lack the adequate knowledge about the emergency management of avulsion [3, 7–10]. Unfortunately, to my knowledge, no study of this type has been conducted in Saudi Arabia so far on the knowledge of ER physicians about the management of tooth avulsion. Therefore, the purposes of this study were to evaluate the knowledge of ER physicians with different specialties, experience and hospital sectors about the management of avulsed teeth in the emergency rooms of eight major hospitals in Jeddah, Saudi Arabia and their attitude toward receiving further education on the emergency management of tooth avulsion.

## Methods

The present study is a cross-sectional survey conducted from August to November 2015. Ethical approval was obtained from local ethical committee (#037–15). The study targeted physicians that were working in the ER department with the following criteria; ER physicians (Consultant or Specialist), pediatricians (Consultant or Specialist), residents having ER rotations in (ER, Pediatrics, or General Surgery), general practitioner assigned in the ER, and medical interns having ER rotations.

Convenient sample was collected from the emergency rooms of six major governmental hospitals and two military hospitals in Jeddah. These were; King Faisal Specialist Hospital, King Abdulaziz University Hospital, King Fahad General Hospital, Al-Thagher Hospital, Al-Aziziyah Maternity and Children Hospital, Jeddah Maternity and Children Hospital, King Fahad Armed Forces Hospital, and the National Guard Hospital. These hospitals were selected because they have an ER department that accept trauma patients.

A self-administered questionnaire with close-ended questions modified from previous studies was used [5, 6, 11, 12]. The questionnaire consisted of 31 questions divided into three parts; 6, 21 and 4 questions in each part respectively. The first part had 6 questions which were about demographic data of the participating physicians, the second part included 21 questions about knowledge related to avulsed tooth and its emergency management, and the last part had 4 questions to evaluate the physician’s attitudes towards learning and receiving more information in order to properly manage such injuries [see Additional file 1]. Confidentiality was maintained, as the questionnaire did not require names or contact numbers of the participants. A consent form was attached to the questionnaire explaining the purpose of the study. It was stated that the participation in survey is voluntary and anyone can choose not to participate in the research or exit the survey at any time without penalty and the information provided by the participant in the questionnaire and the results of this study will be used for scholarly purposes only. Also, it was mentioned in the consent that; by answering the questionnaire questions the participant is fully understand the contents of this document and is agreeing to participate in this study.

A total of 150 questionnaires were distributed and the physician’s participation was voluntarily. Twenty-eight physicians refused to participate and complete their questionnaires because they were exhausted or busy. Two investigators had the responsibility of distributing and collecting the questionnaires by themselves, in order to ensure its completion process without involving colleagues’ consultation.

Data were collected and statistically analyzed using Mann-Whitney U, Kruskal-Wallis H, and Bonferroni tests (SPSS program version 20, SPSS, Chicago, IL, USA).

## Results

### Demographic data (Table 1)

Out of 150 ER physicians in all eight hospitals, 122 responded (81.33%). The majority of them were male (68.9%). The minimum age of the participants was 25 years and the maximum was 55 years. 74.6% of the participants were between 20 and 39 years old. They were 16 consultants (13.1%), 24 specialists (19.7%), 16 general practitioners (13.1%), 46 residents (37.7%), and 20 interns (16.4%). Their specialties were; ER (44.2%), Pediatrics (32.8%), General Surgery (6.6%), and interns (16.4%). 50.8% of them were Saudi and 49.2% were non-Saudi.

**Table 1:** Demographic data and knowledge level of the participants

Demographic data			Knowledge Level		
Variables		*N* (%)	Mean(SD)	Median	*P* value
Gender	Male	84 (68.9)	61.9 (21.3)	66.7	0.234
	Female	38 (31.1)	58.5 (20.4)	61.1	
Age	20- < 30	45 (36.9)	46.1 (17.7)	41.7	0.002
	30- < 40	46 (37.7)	67.6 (17.3)	72.2	
	40- < 50	19 (15.6)	69.8 (17.3)	77.8	
	≥50	12 (9.8)	87.5 (4.9)	86.1	
Nationality	Saudi	62 (50.8)	57.3 (24.0)	61.1	0.165
	Non-Saudi	60 (49.2)	64.4 (16. 8)	66.7	
Specialty	ER	54 (44.2)	69.7 (16.8)	77.8	0.000
	Pediatric	40 (32.8)	59.7 (20.8)	61.1	
	General Surgery	8 (6.6)	45.8 (19.9)	50	
	Intern	20 (16.4)	38.9 (15.2)	38.9	
Experience Level (occupation)	Consultant	16 (13.1)	82.6 (5.3)	83.3	0.000
	Specialist	24 (19.7)	70.4 (13.2)	72.2	
	Resident	46 (37.7)	51.6 (19.6)	55.6	
	General Practitioner	16 (13.1)	73.6 (14.6)	72.2	
	Intern	20 (16.4)	42 (16.9)	38.9	
Previous exposure to avulsion cases	Yes	72 (59)	67.6 (17.67)	72.22	0.000
	No	50 (41)	51.1 (21.76)	50	
Hospitals	Public	62 (50.8)	56.6 (19.2)	55.6	0.009
	Military	60 (49.2)	65.2 (22)	72.2	

### Previous knowledge and experience with dental avulsion (Table 2)

Data revealed that 47.5% of the respondents did not have prior knowledge regarding the subject. Medical books (20.5%) and residency programs (15.6%) were the two major sources of information to those who had previous knowledge. With reference to the meaning of tooth avulsion, 68.9% of the participants knew the correct meaning and 59% of them mentioned that they came across avulsion cases (Table 2).

**Table 2:** Previous knowledge and experience with dental avulsion

Questions	Options	Frequency	Percent
What is tooth avulsion?	Total dislodgement of intact tooth out of its socket due to any trauma	84	68.9
	Dislodgement of fractured segment of the tooth due to any trauma	17	14
	Tooth fracture due to any trauma	5	4.1
	Don’t know	16	13
Do you have any prior knowledge about the management of avulsed tooth?	Yes	64	52.5
	No	58	47.5
If yes, what was your source of information?	Residency program	19	15.6
	Health talks on TV or radio	7	5.7
	Medical books	25	20.5
	Others	13	10.7
	NA^a^	58	47.5
Have you ever come across a patient with avulsed tooth (knocked out)?	Yes	72	59.0
	No	50	41.0

^a^*NA* answered (No) to the previous question

### Knowledge about re-implantation and extra-oral time (Table 3)

Most of the physicians (87.7%) responded that they would refer or instruct the parent to visit the dentist after avulsion, however, 54% of them would advise the parents to go immediately. When participants were asked about the possibility of replanting the tooth, 79.5% answered positively, however, 27.9% of them would do it by themselves. Furthermore, slightly over half of the physicians (51.6%) knew the importance of extra-oral time (Table 3).

**Table 3:** Knowledge about re-implantation and extra-oral time

Questions	Options	Frequency	Percent
Did you or would you refer the child or instruct the parents to go to the dentist after avulsion?	Yes	107	87.7
	No	15	12.3
If YES, When will you advise the parents to go to the dentist?	Immediately	66	54.0
	Next day	23	18.8
	After few days, when child is comfortable	2	1.6
	Only if any pain or other symptoms are noticed	13	10.6
	NA^a^	15	12.0
	Missing	4	3.0
Do you think that the avulsed permanent tooth can be put back?	Yes	97	79.5
	No	25	20.5
If YES, will you do it by yourself?	Yes	34	27.9
	No	63	51.6
	NA^a^	25	20.5
Is extra oral time is important?	Yes	63	51.6
	No	6	4.9
	Don’t know	53	43.5

^a^*NA* answered (No) to the previous question

### Handling, cleaning and transport media (Table 4)

In relation to tooth handling, 51.6% of physicians would hold the avulsed tooth from the crown. Almost half of the physicians (48.4%) chose to rinse the tooth under running water if it was covered with soil before replanting it.

**Table 4:** Knowledge about tooth handling, cleaning and transport media

Questions	Options	Frequency	Percent
If you decided to replant the tooth into its socket, but it has fallen onto the ground and is covered with dirt, what would you do?	Rinse tooth under running water	59	48.4
	Gently wipe off the dirt that is stuck to the tooth by hand	4	3.3
	Scrub the tooth gently with a toothbrush	2	1.6
	Spray alcohol on the tooth	15	12.3
	Put the tooth straight back into the socket, with no pretreatment	2	1.6
	Don’t know	40	32.8
If you did not replant the tooth, How would you carry the tooth to the dentist?	Wrap in paper or gauze	26	21.3
	Pack the tooth in ice	7	5.7
	Put in water	10	8.2
	Put in milk	38	31.1
	Put in saline	26	21.3
	Put in alcohol	3	2.5
	Put in disinfecting solution	5	4.2
	Hold the tooth in the child’s mouth	0	0
	Missing	7	5.7
How to hold an avulsed tooth?	From the crown	63	51.6
	From the root	2	1.6
	Anywhere (crown or root)	11	9.1
	Don’t know	46	37.7

Milk was selected as the best transport media for avulsed tooth by 31.1% of the participants, followed by saline (21.3%). However, 21.3% of the physicians decided to wrap the tooth in paper or gauze (Table 4).

### Physicians attitude (Table 5)

Most of the participants (63.1%) believed that they have an inadequate level of information about traumatic dental injuries. Accordingly, 95.1% of them had shown interest in receiving more information in order to properly manage such injuries and all of them (100%) agreed on the importance of learning about this subject (Table 5).

**Table 5:** Physicians Attitude

Questions	Options	Frequency	Percent
What do you think is your level of information about traumatic dental injuries?	Adequate	29	23.8
	Inadequate	77	63.1
	Don’t know	16	13.1
In your opinion, learning about traumatic dental injuries is:	Very important	27	22.1
	Important	95	77.9
	Somewhat important	0	0
	Not important	0	0
Would you like to receive more information in order to properly manage traumatic dental injuries?	yes	116	95.1
	no	6	4.9
Are you interested in knowing the emergency management of avulsed tooth?	yes	116	95.1
	no	6	4.9

### Groups’ comparisons on knowledge level (Table 1)

Data were not normally distributed; therefore nonparametric tests were used (Table 1).

To compare mean knowledge of different physicians’ gender, nationality, previous exposure to avulsion cases and different hospital sectors, Mann-Whitney U test was used. Results showed that there was no statistical significant difference between male and female (*p* = 0.234), nor between Saudi and non-Saudi Physicians (*p* = 0.165). However, there was a statistical significant difference between physicians with previous exposure to avulsion cases and those with no experience (*p* = 0.000). Physicians who came across avulsion cases had better knowledge about the ER management of such cases than those with no experience. Moreover, there was a statistical significant difference between physicians working in military and public hospitals (*p* = 0.009). Results showed that physicians working in military hospitals had better knowledge about the ER management of avulsion cases than those working in public hospitals.

To compare mean knowledge of different physicians’ age, specialty, and experience level, Kruskal-Wallis H test was used. Results showed that there was a statistical significant difference between different age groups (*p* = 0.002), different specialties (*p* = 0.000), and different experience levels (*p* = 0.000).

### Comparisons within the groups (Table 6), (Table 7) and (Table 8)

To compare mean knowledge of physicians within the groups, Bonferroni test was used at 5% alpha error. There was a statistical significant difference between all age groups except between (30- < 40) and (40- < 50), and between (40- < 50) and (≥50) groups (Table 6).

**Table 6:** Bonferroni test for different age groups

(I) Age	(J) Age	Mean Difference (I-J)	Std. Error	Sig.	95 % Confidence Interval	
					Lower Bound	Upper Bound
20–29	30–39	−21.56804^*^	3.50163	.000	−30.9673	−12.1688
	40–49	−23.68827^*^	4.69053	.000	−36.2788	−11.0977
	> 50	−41.43519^*^	6.48089	.000	−58.8316	−24.0388
30–39	20–29	21.56804^*^	3.50163	.000	12.1688	30.9673
	40–49	−2.12024	4.71825	1.000	−14.7852	10.5448
	> 50	−19.86715^*^	6.50099	.017	−37.3175	−2.4168
40–49	20–29	23.68827^*^	4.69053	.000	11.0977	36.2788
	30–39	2.12024	4.71825	1.000	−10.5448	14.7852
	> 50	−17.74691	7.21127	.092	−37.1038	1.6100
> 50	20–29	41.43519^*^	6.48089	.000	24.0388	58.8316
	30–39	19.86715^*^	6.50099	.017	2.4168	37.3175
	40–49	17.74691	7.21127	.092	−1.6100	37.1038

**Table 7:** Bonferroni test for different specialty

Specialty	Specialty	Mean Difference	Std. Error	Sig.	95% Confidence Interval
					Lower Bound
ER	Pediatric	10.11905	3.83961	.057	−.1844
	GS	24.00794^*^	7.01014	.005	5.1965
	Intern	27.86596^*^	5.02529	.000	14.3808
Pediatric	ER	−10.11905	3.83961	.057	−20.4225
	GS	13.88889	7.18325	.333	−5.3871
	Intern	17.74691^*^	5.26409	.006	3.6209
GS	ER	−24.00794^*^	7.01014	.005	−42.8194
	Pediatric	−13.88889	7.18325	.333	−33.1649
	Intern	3.85802	7.88099	1.000	−17.2903
Intern	ER	−27.86596^*^	5.02529	.000	−41.3512
	Pediatric	−17.74691^*^	5.26409	.006	−31.8729
	GS	−3.85802	7.88099	1.000	−25.0064

*The mean difference is significant at the 0.05 level

**Table 8:** Bonferroni test for different experience level (occupation)

Occupation	Occupation	Mean Difference	Std. Error	Sig.	95% Confidence Interval
					Lower Bound
Consultant	Specialist	12.26852	5.21165	.202	−2.6433
	Resident	31.01852^*^	4.66144	.000	17.6810
	GP	9.02778	5.70907	1.000	−7.3073
	Intern	40.66358^*^	5.54822	.000	24.7888
Specialist	Consultant	−12.26852	5.21165	.202	−27.1803
	Resident	18.75000^*^	4.03693	.000	7.1994
	GP	−3.24074	5.21165	1.000	−18.1525
	Intern	28.39506^*^	5.03493	.000	13.9889
Resident	Consultant	−31.01852^*^	4.66144	.000	−44.3560
	Specialist	−18.75000^*^	4.03693	.000	−30.3006
	GP	−21.99074^*^	4.66144	.000	−35.3283
	Intern	9.64506	4.46299	.327	−3.1246
GP	Consultant	−9.02778	5.70907	1.000	−25.3628
	Specialist	3.24074	5.21165	1.000	−11.6711
	Resident	21.99074^*^	4.66144	.000	8.6532
	Intern	31.63580^*^	5.54822	.000	15.7610
Intern	Consultant	−40.66358^*^	5.54822	.000	−56.5384
	Specialist	−28.39506^*^	5.03493	.000	−42.8012
	Resident	−9.64506	4.46299	.327	−22.4148
	GP	−31.63580^*^	5.54822	.000	−47.5106

*The mean difference is significant at the 0.05 level

In relation to different specialties, the highest mean knowledge was the ER physicians followed by pediatric physicians. There was a statistical significant difference between interns and the following two groups; the ER (*p* = 0.00) and pediatric physicians (*p* = 0.006) as well as between the general surgeons and ER physicians (*p* = 0.005) (Table 7).

In relation to the experience level of physicians (occupation), the highest mean knowledge was of the consultants, followed by the general practitioners (GPs), then specialists. The lowest mean knowledge was of the interns followed by residents. There was a statistical significant difference between residents and the following senior physicians’ groups; consultants (*p* = 0.00), specialists (*p* = 0.00) and GPs (*p* = 0.00) as well as between interns and; consultants (*p* = 0.00), specialist (*p* = 0.00) and GPs (*p* = 0.00) (Table 8).

## Discussion

This study provided a baseline information about the current level of knowledge of ER physicians about tooth avulsion. The results of the survey showed that nearly a third of physicians (31.1%) did not know what an avulsed tooth was. This result was lower than Dali et al. [11] result where more than half of their participants did not know the meaning of tooth avulsion. Medical books and residency programs were the two major sources of knowledge for those who had previous information about the subject. Moreover, 59% of the participants came across avulsion cases. However, 47.5% of them had no previous knowledge about its management. This result was in agreement with several previous studies done in Kuwait, USA, and Nepal where the percentage were 83.3%, 67%, and 78.9% respectively [9, 11, 13]. These results reflect the importance of this study, since ER physicians are the first to contact and provide primary care for those cases.

Interestingly, there was a statistical significant difference in the level of knowledge between physicians working in military and public hospitals. Results showed that physicians working in military hospitals had better knowledge than those working in public hospitals. This might be attributed to the fact that military hospitals have more trauma cases than the other hospitals. However, this area needs further investigation. Moreover, there was a statistical significant difference between different age groups, different experience level and previous exposure to avulsion cases. All these differences are understandable and could be explained that with age, experience, as well as the exposure to avulsion cases, knowledge about ER management of avulsed tooth would increase.

The ideal treatment of tooth avulsion that determines long-term success is immediate replantation. The majority of the participants (79.5%) thought it was possible to replant the avulsed tooth. However, 51.6% of them would not do it by themselves. This result was higher than Hashim’s results [12] which showed that none of the participants preferred to put the tooth back into the socket before referring to the dentist. Similarly, several studies showed that the majority of participants would prefer to refer the patient to the dentist because they felt that those cases should be managed by dentists [6, 8, 11]. This was in agreement with the result of this study where 87.7% of the participants would refer or instruct the parents to visit the dentist after avulsion. However, 54% of them would advise the parents to go immediately. This was in consensus with Hashim’s results [12] where 52% of the physicians recognized the importance of immediate dental treatment. These results further indicated the needs and the importance of education since prognosis would be much better with immediate and appropriate treatment.

In relation to tooth handling, 51.6% of the physicians knew that the tooth should be held by the crown. This result was in agreement with Dali et al. [11] (50%) and less than Jyothi et al. [6] (72.8%). Furthermore, almost half of the physicians (48.4%) chose to rinse the tooth under running water if it was covered with soil. Our results were less than Dali et al. [11] (84.7%) and Jyothi et al. [6] (77.14%) where most of their participants were aware that the tooth should be cleaned gently under running water to avoid damage to the periodontal cells.

Another factor that affects the prognosis and the success of treatment is the extra-oral time. Any delay in seeking professional treatment will jeopardize the prognosis. The optimum time for replantation is between 15 and 20 min. In this study, 48.4% of the physicians did not know the importance of the extra-oral time. This was in consensus with other studies’ results [6, 11].

If replantation is not feasible, the use of a correct transport media is an essential step, which will expand the extra-oral time to 1 h. Transport media are used to maintain the periodontal ligament cells’ viability, increase their survival and prevent any damage which might cause loss of the tooth in the future as ankylosis and resorption.

Theoretically, the best media for that function are HBSS, ViaSpan and Eagle’s medium. However, they are not used because of their cost and unavailability for everyone. Practically, Milk is considered the best medium because of its convenience, cost and availability as well as its ability to preserve PDL cells viability [3, 6]. It is an isotonic liquid with favorable osmolality, which has essential nutrients for periodontal ligament cells to survive and allow them to heal [6, 7]. Therefore, it has been recommended as a temporary storage medium for avulsed teeth before replantation [14]. In our study, 31.1% of the participants chose milk as the best medium for storing the tooth. This result was similar to Ulusoy et al. [5], where 31.9% of the participants also chose milk as the best medium. However, our result was greatly higher than other studies [11, 12].

If replantation is not feasible, the use of a correct transport media is an essential step, which will expand the extra-oral time to 1 h. This will maintain the periodontal ligament cells’ viability and prevent any damage which might cause loss of the tooth in the future. Milk is considered the best medium for the periodontal ligament cells viability [3, 6]. It is an isotonic liquid with favorable osmolality, which has essential nutrients for periodontal ligament cells to survive and allow them to heal [6, 7]. Therefore, it has been recommended as a temporary storage medium for avulsed teeth before replantation [14]. In our study, 31.1% of the participants chose milk as the best medium for storing the tooth. This result was similar to Ulusoy et al. [5], where 31.9% of the participants also chose milk as the best medium. However, our result was greatly higher than other studies [11, 12].

Another aspect of this study was the participants’ attitude towards learning about this subject. All the participants (100%) agreed on the importance of learning about traumatic dental injuries. More than half of the participants (63.1%) believed that they do not have adequate level of information about traumatic dental injuries. The majority of the participants (95.1%) were keen to learn more about the management of avulsed teeth. Our results were in consensus with Ulusoy et al. [5] where 78.3% of the participants felt that they need further education about the subject.

## Conclusion

The study findings showed that the majority of the ER physicians lack the knowledge needed to manage avulsion cases. The results stressed on the importance and the need of educating ER physicians about the emergency management of avulsed teeth. Traumatic dental injuries and the ER management should be integrated in the under graduate curriculum and post graduate programs to graduate physicians with an appropriate level of knowledge to manage ER cases.

## Additional file

Additional file 1: The questionnaire used in the study. (PDF 214 kb)

## Abbreviations

ER: Emergency room
GP: General practitioner
GS: General surgeon
HBSS: Hank’s Balanced Salt Solution
PDL: Periodontal ligament
