# Supplementary material for: Knowledge and attitude of emergency physician about the emergency management of tooth avulsion
Source: BMC Oral Health. 2018 Apr 2;18:57. doi: 10.1186/s12903-018-0515-5 (PMC5879752; doi:10.1186/s12903-018-0515-5)
Supplement: Supplementary file 1 — The questionnaire used in the study. (PDF 214 kb) [file 12903_2018_515_MOESM1_ESM.pdf]

Serial no……..……….

Traumatic Dental Injuries

1- Gender

a- Male b- Female

2- Age

a- 20-29 b- 30-39 c- 40-49 d- > 50

3- Place of work …………………………..

4- Occupation ……………………………..

5- Specialty ………………………………..

6- Nationality ……………………………..

7- What is avulsion of a tooth?

a. Total dislodgement of intact tooth out of its socket due to any trauma
b. Dislodgement of fractured segment of the tooth due to any trauma
c. Tooth fracture due to any trauma
d. Gum bleeding due to any trauma
e. Don't know

8- Do you have any prior knowledge about the management of avulsed tooth?

a- Yes
b- No

9- If yes what was your source of information?

a. Residency program.
b. Health talks on television or radio.
c. Conference/Convention.
d. Medical books.
e. Others (specify)……………………………….

10- Have you ever come across a patient with avulsed tooth (knocked out)?

a- Yes
b- No

11- Did you or would you refer the child or instruct the parents to go to the dentist after avulsion?

a- Yes
b- No

12- If YES, When will you advise the parents to go to the dentist?

a- Immediately
b- Next day
c- After few days, when the child is comfortable
d - Only if any pain or other symptoms are noticed

13- A boy came to you with a knocked-out permanent anterior tooth in his hand after the accident. What will you do with the tooth?

a- Save
b- Discard
c- Put the tooth back into the socket (its place)

14- If you decided to save the tooth, what will you do with it?

a- Wash with water/ other liquid
b- Clean it with a tissue paper
c- Nothing (leave it as it is)

If you were at a site where a child knocked-out his permanent front tooth.

15- Would you advise the parents to look for the avulsed tooth?

a- Yes
b- No

16- Do you think it is necessary to save avulsed permanent tooth?

a- Yes
b- No

17- Do you think that the avulsed permanent tooth can be put back?

a- Yes
b- No

18- If YES, will you do it by yourself?

a- Yes
b- No

19- If you decided to replant the tooth into its socket, but it has fallen onto the ground and is covered with dirt, what would you do?

a- Rinse the tooth under running water.
b- Gently wipe off the dirt that is stuck to the tooth by hand.
c- Scrub the tooth gently with a toothbrush.
d- Spray alcohol on the tooth.
e- Put the tooth straight back into the socket, with no pretreatment.
f- Don’t know/other ………………………………….

20- What would you do if the tooth was in child’s mouth, however, out of place?

a- Put the tooth back into the socket (its place)
b- Leave the tooth inside the mouth
d- Remove the tooth outside the mouth

21- If you did not replant the tooth, would you advise the parents to take the tooth to the dentist?

a- Yes
b- No

22- Is extra oral time is important?

a- Yes
b- No
c- Don’t know

23- When should the tooth be put back, if it had been knocked out of the mouth?

a- Immediately
b- As soon as the bleeding has stopped
c- During the first hour
d- Within the same day
e- After few days
f- When visiting the dentist
g- Don't know

24- If you did not replant the tooth, How would you carry the tooth to the dentist?

a- Wrap in paper or gauze
b- Pack the tooth in ice
c- Put in Water
d- Put in Milk
e- Put in Saline
f- Put in alcohol
f- Hold the tooth in the child's mouth.
h- Put in disinfecting solution

25- How to hold an avulsed tooth?

a- From the crown
b- From the root
c- Anywhere (crown or root)
d- Don't know

26- Can you differentiate if the tooth is primary or permanent?

a. Yes
b. No

27- Would you care if the tooth that has been knocked-out was a primary tooth?

a. Yes
b. No
c. Don't know

28- What do you think is your level of information about traumatic dental injuries?

a- Adequate
b- Inadequate
c- Don't know

29- In your opinion, learning about traumatic dental injuries is:

a- Not important
b- Important
c- Somewhat important
d- Very important

30- Would you like to receive more information in order to properly manage traumatic dental injuries?

a- Yes
b- No

31- Are you interested in knowing the emergency management of avulsed tooth?

a- Yes
b- No
